# MACRPO: Multi-agent cooperative recurrent policy optimization

**DOI:** 10.3389/frobt.2024.1394209

**Published:** 2024-12-20

**Authors:** Eshagh Kargar, Ville Kyrki

**Affiliations:** Intelligent Robotics Group, Electrical Engineering and Automation Department, Aalto University, Helsinki, Finland

**Keywords:** cooperative, policy, multi-agent, information sharing, interaction, reinforcement learning

## Abstract

This work considers the problem of learning cooperative policies in multi-agent settings with partially observable and non-stationary environments without a communication channel. We focus on improving information sharing between agents and propose a new multi-agent actor-critic method called *Multi-Agent Cooperative Recurrent Proximal Policy Optimization* (MACRPO). We propose two novel ways of integrating information across agents and time in MACRPO: First, we use a recurrent layer in the critic’s network architecture and propose a new framework to use the proposed meta-trajectory to train the recurrent layer. This allows the network to learn the cooperation and dynamics of interactions between agents, and also handle partial observability. Second, we propose a new advantage function that incorporates other agents’ rewards and value functions by controlling the level of cooperation between agents using a parameter. The use of this control parameter is suitable for environments in which the agents are unable to fully cooperate with each other. We evaluate our algorithm on three challenging multi-agent environments with continuous and discrete action spaces, Deepdrive-Zero, Multi-Walker, and Particle environment. We compare the results with several ablations and state-of-the-art multi-agent algorithms such as MAGIC, IC3Net, CommNet, GA-Comm, QMIX, MADDPG, and RMAPPO, and also single-agent methods with shared parameters between agents such as IMPALA and APEX. The results show superior performance against other algorithms. The code is available online at https://github.com/kargarisaac/macrpo.

## 1 Introduction

While reinforcement learning (RL) (Kaelbling et al., 1996) has gained popularity in policy learning, many problems that require coordination and interaction between multiple agents cannot be formulated as single-agent reinforcement learning. Examples of such scenarios include self-driving cars (Shalev-Shwartz et al., 2016), autonomous intersection management (Dresner and Stone, 2008), multiplayer games (Berner et al., 2019; Vinyals et al., 2019), and distributed logistics (Ying and Dayong, 2005). Solving these kinds of problems using single-agent RL is problematic because the interaction between agents and the non-stationary nature of the environment due to multiple learning agents can not be considered (Hernandez-Leal et al., 2019; Lazaridis et al., 2020). Multi-agent reinforcement learning (MARL) and cooperative learning between several interacting agents can be beneficial in such domains and has been extensively studied (Nguyen et al., 2020; Hernandez-Leal et al., 2019).

However, when several agents are interacting with each other in an environment without real-time communication, the lack of communication deteriorates policy learning. To alleviate this problem, we propose a meta-trajectory-based communication scheme during training, where agents indirectly share information through a centralized critic. By aggregating trajectories from all agents into a meta-trajectory, the critic is able to learn the cooperative dynamics between agents, even in the absence of direct communication during execution. This scheme enables the agents to implicitly learn from each other’s observations and rewards, allowing them to better predict each other’s behavior and adapt accordingly. For example, in applications like autonomous driving at intersections, agents can anticipate the actions of others, improving performance, safety, and cooperation.

A standard paradigm for multi-agent planning is to use the centralized training and decentralized execution (CTDE) approach (Kraemer and Banerjee, 2016; Foerster et al., 2016; Lowe et al., 2017; Foerster et al., 2018; Xiao et al., 2021), which we also adopt in this work. During centralized training, the critic receives global information, including observations and actions from all agents, allowing it to model the inter-agent dynamics. During decentralized execution, each agent uses its own local observations to act independently.

**FIGURE 1:**
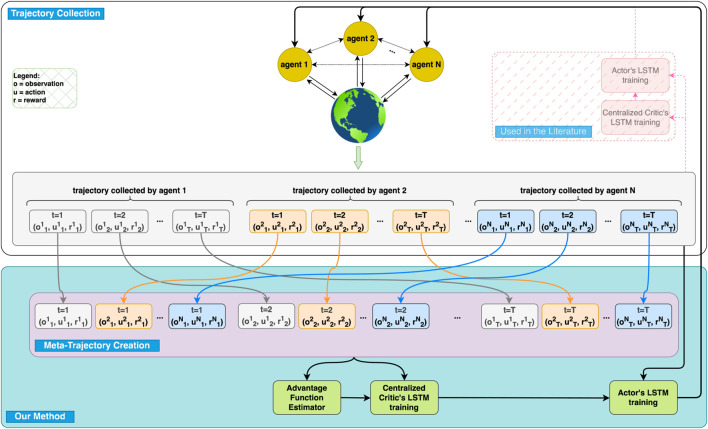
Overview of the Multi-Agent Cooperative Recurrent Proximal Policy Optimization (MACRPO) Framework. This figure illustrates the key components of our proposed method (shown with full opacity) compared to conventional approaches in the literature (shown with transparency). In our method, each agent collects trajectories consisting of observations (o), actions (u), and rewards (r) over multiple time steps. These individual trajectories are then combined into a meta-trajectory, which is fed into both the advantage function estimator and the centralized critic’s LSTM. The advantage function estimator (added in our method) calculates each agent’s advantage by considering both individual and shared rewards, thereby allowing us to control the cooperation level between agents. This calculated advantage helps the centralized critic’s LSTM to better learn the dynamics and cooperation between agents. During decentralized execution, only the actor networks are used, trained independently for each agent. In contrast, conventional approaches use separate trajectories and do not incorporate meta-trajectory or cooperative advantage estimation.

In this work, we propose a new cooperative multi-agent reinforcement learning algorithm, which is an extension to Proximal Policy Optimization (PPO), called *Multi-Agent Cooperative Recurrent Proximal Policy Optimization* (MACRPO). MACRPO enhances inter-agent coordination through two key mechanisms: First, it uses a recurrent long short-term memory (LSTM) layer in the critic network, trained with a meta-trajectory that combines trajectories from all agents (see Figure 1). This enables the critic to capture the temporal dynamics and interactions between agents over time, while also handling the partial observability of each agent. Second, MACRPO introduces a novel advantage function estimator that incorporates both the rewards and value functions of other agents, controlled by a cooperation parameter. This allows MACRPO to adjust the level of cooperation, which is particularly useful in environments where agents cannot fully cooperate, balancing individual and collective rewards.

**FIGURE 2:**
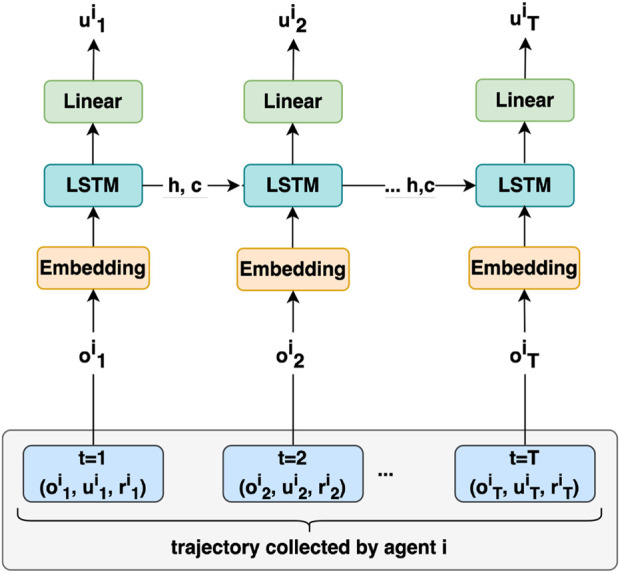
Actor Network Architecture for agent i . The actor network processes its own collected trajectory, consisting of local observations $\left(o_{i}^{t}\right)$ and actions $\left(u_{i}^{t}\right)$ over time. It outputs the agent’s action at each time-step based on its observations and learned policy.

MACRPO operates under the centralized training and decentralized execution paradigm. During training, the centralized critic leverages the meta-trajectory to sequentially predict the value of a state for each agent, enabling it to learn the cooperative strategies between agents. During execution, the decentralized actor networks use only local observations, ensuring that each agent acts autonomously without requiring real-time communication.

Moreover, in environments where multiple agents are simultaneously learning during training, each agent’s policy and the environment’s dynamics are constantly changing from the perspective of other agents, resulting in non-stationarity (Hernandez-Leal et al., 2019; Xiao et al., 2021). To mitigate this issue, MACRPO employs an on-policy approach, ensuring that the most recent data collected from the environment is used for training.

In summary, our contributions are as follows: (1) We propose a cooperative on-policy centralized training and decentralized execution framework that is applicable to both discrete and continuous action spaces. (2) We introduce two novel mechanisms for information sharing across agents: (a) a recurrent LSTM component in the network architecture that integrates meta-trajectories to capture inter-agent cooperation over time, and (b) an advantage function estimator that combines individual rewards and value functions with a control parameter, which allows the level of cooperation between agents to be dynamically adjusted. This dual mechanism enables superior policy learning and adaptability compared to prior approaches, as demonstrated in our experiments. (3) We evaluate MACRPO on three cooperative multi-agent tasks: DeepDrive-Zero (Quiter, 2020), Multi-Walker (Gupta et al., 2017), and Particle (Mordatch and Abbeel, 2018), showing that it achieves comparable or superior performance against state-of-the-art methods.

The rest of this paper is organized as follows. The review of related works in Section 2 demonstrates that while MARL has been extensively studied, existing approaches do not address the dynamics of interaction between agents in detail. In Section 3, we provide the required background in Markov Games and Proximal Policy Optimization. The problem definition and the proposed method are described in Section 4, with emphasis on the two innovations, meta-trajectory for recurrent network training and joint advantage function. Then, Section 5 presents empirical evaluation in three multi-agent environments showing superior performance of the proposed approach compared to the state-of-the-art. Finally, in Section 6 we conclude that implicit information sharing can be used to improve cooperation between agents while discussing its limitations in settings with a high number of agents.

## 2 Related work

The most straightforward and maybe the most popular approach to solving multi-agent tasks is to use single-agent RL and consider several independent learning agents. Some prior works compared the performance of cooperative agents to independent agents and tried independent Q-learning (Tan, 1993) and PPO with LSTM layer (Bansal et al., 2017), but they did not work well in practice (Matignon et al., 2012). Also, Zhao et al. (2020) tried to learn a joint value function for two agents and used PPO with an LSTM layer to improve the performance in a multi-agent setting.

In order to use single-agent RL methods for multi-agent settings, improve the performance, and speed up the learning procedure, some works used parameter sharing between agents (Gupta et al., 2017; Terry et al., 2020). Especially in self-play games, it is common to use the current or older versions of the policy for other agents (Berner et al., 2019). We will compare our proposed method with several state-of-the-art single-agent RL approaches with shared parameters between agents proposed in Terry et al. (2020) in the experiments section. Our way of training the LSTM layer in the critic differs from parameter sharing used in the literature such that instead of using separate LSTMs for each agent, the LSTM layer in our method has a shared hidden state, which is updated using a combination of all agents’ information. This lets the LSTM layer learn about the dynamics of interaction and cooperation between agents across time.

In addition to using single-agent RL methods with or without parameter sharing, some works focus on designing multi-agent RL algorithms that incorporate communication between agents to enhance coordination in multi-agent settings. Communication in multi-agent environments can significantly improve learning and performance by enabling agents to share information (Niu et al., 2021; Singh et al., 2019; Liu et al., 2020; Sukhbaatar et al., 2016; Dutta et al., 2005; Da Silva and Costa, 2019; Kash et al., 2011). However, the effectiveness of communication often depends on the availability and optimization of the communication channels. IC3Net (Singh et al., 2019) introduces a communication gating mechanism, allowing agents to learn when to communicate with each other during cooperative and competitive tasks. While IC3Net leverages communication between agents to improve coordination, it assumes that a communication channel is available during execution. In some real-world scenarios, such as autonomous driving, direct communication between agents may not be feasible. In such environments, agents must learn to cooperate without explicit communication. Unlike IC3Net, MACRPO addresses this challenge by enabling indirect information sharing during the training phase through a meta-trajectory that combines the experiences of all agents. This meta-trajectory allows agents to implicitly learn cooperation strategies without requiring explicit communication during execution, making MACRPO well-suited for environments where communication is restricted or absent.

A recently popularized paradigm for sharing information between agents is the use of centralized training and decentralized execution. In general, we can categorize these types of approaches into two groups: value-based and actor-critic-based. In value-based methods, the idea is to train a centralized value function and then extract the value functions for each agent from that to act in a decentralized manner in the execution time (Sunehag et al., 2018; Rashid et al., 2018). On the other hand, the actor-critic-based methods have actor and critic networks (Lowe et al., 2017; Foerster et al., 2018). The critic network has access to data from all agents and is trained in a centralized way, but the actors have only access to their local information. They can act independently in the execution time. The actors can be independent with individual weights (Lowe et al., 2017) or share the policy with shared weights (Foerster et al., 2018). In this work, we use an actor-critic-based method with centralized training and decentralized execution, providing two innovations to improve information sharing without a communication channel between agents during execution.

RMAPPO (Yu et al., 2022) is a method close to ours, which uses the CTDE framework. They make no mention of recurrent neural networks (RNNs) in their paper, but their code contains recurrent layers. RMAPPO focuses primarily on adapting PPO components such as clipping, mini-batching, batch size, value normalization, and value function input representation for multi-agent environments. However, the main distinction between our work and theirs lies in the meta-trajectory we generate from the data of all agents, and the specific manner in which we employ the RNN layer. RMAPPO employs CTDE and RNNs as usual without a combined trajectory as input, which limits its ability to model interactions between agents over time. In addition to the meta-trajectory, another difference is in the way information is shared. While both methods share policy parameters across agents, RMAPPO uses a shared reward function for all agents that is the sum of individual rewards, without any control over the degree of cooperation. In contrast, MACRPO introduces a novel advantage function estimator that incorporates a control parameter, allowing us to dynamically adjust the level of cooperation between agents. This provides greater flexibility in environments where the degree of cooperation must vary dynamically over time. Furthermore, while RMAPPO is tested primarily in environments with discrete action spaces, MACRPO is evaluated in both discrete and continuous action spaces, demonstrating its broader applicability.

In Foerster et al. (2018), which is another work near ours, the actor is recurrent, but the critic is a feed-forward network, whereas our actor and critic are both recurrent, and the recurrent layer in our critic has a crucial role in our method. Their method is also for settings with discrete action spaces, whereas we test our method on three environments with both discrete and continuous action spaces.

ROLA (Xiao et al., 2021) is another work near ours. They use LSTMs in both actor and critic networks. Additionally, ROLA employs both centralized and individual asymmetric critics that estimate individual advantage values using local history and/or state information. However, we construct the meta-trajectory which has not only the history of each agent but also the history of the interaction between agents and the environment’s dynamics. In addition, we propose a novel advantage function estimator which is a combination of all agents’ advantage functions and the cooperation level of agents can be changed based on the problem using a control parameter.

Durugkar et al. (2020) is also a work that combines an agent-specific reward and an environment-specific reward to accomplish the shared task. They consider a framework that uses a linear mixing scheme to balance individual preferences and task rewards. They demonstrate that in their test environments, a small amount of selfishness and not full cooperation can be advantageous and facilitate team learning. In our test environments and with our framework, full cooperation among agents yields superior performance. Depending on the environment, the amount of cooperation and selfishness can be different.

The other similar work to ours, which is one of the most popular MARL methods, is the multi-agent deep deterministic policy gradient (MADDPG) (Lowe et al., 2017) that proposed similar frameworks with centralized training and decentralized execution. They tested their method on some Particle environments (Mordatch and Abbeel, 2018). Their approach differs from ours in the following ways: (1) They do not have the LSTM (memory) layer in their network, whereas the LSTM layer in the critic network plays a critical role in our method. It helps to learn the interaction and cooperation between agents and also mitigate the partial observability problem. (2) They tested MADDPG on Multi-Agent Particle Environments with discrete action spaces. However, we test our method in both continuous and discrete action space environments. (3) They consider separate critic networks for each agent, which is beneficial for competitive scenarios, whereas we use a single critic network and consider the cooperative tasks. (4) Their method is off-policy with a replay buffer, and they combat the non-stationarity problem by centralized training. In contrast, our approach, in addition to centralized training, is an on-policy method without a replay buffer allowing the networks to use the most recent data from the environment. We will compare our method with MADDPG and show that ours has comparable or superior performance. Wang et al. (2020) extends the MADDPG idea and adds a recurrent layer into the networks, but they have separate actors and critics for agents, similar to MADDPG, and recurrent hidden states of critics are isolated, and there is no combination of information in them. They also tested their method on one environment with a discrete action space.

We target problems where agents attempt to collaboratively maximize the sum of all agents’ expected rewards but where each agent receives its reward. We do not specifically consider the credit assignment problem for multi-agent games where all agents have a shared team reward. The proposed algorithm can be applied to such problems, but it is not designed for them.

To provide a clearer comparison, Table 1 summarizes the key differences between MACRPO and other state-of-the-art methods. The table highlights the distinctive features of MACRPO, such as the use of meta-trajectories and a novel advantage estimation mechanism with a cooperation control parameter, which are not present in other approaches.

**TABLE 1:** Comparison of MACRPO with state-of-the-art multi-agent reinforcement learning methods.

Method	CTDE framework	Recurrent critic	Meta-trajectory	Advantage estimation with cooperation control	Use of communication	Action space
Independent RL Tan (1993)	✗	✗	✗	✗	None	Discrete, Continuous
Parameter Sharing Gupta et al. (2017); Terry et al. (2020)	✗	✗	✗	✗	None	Discrete, Continuous
IC3Net Singh et al. (2019)	✓	✗	✗	✗	Explicit (Gating)	Discrete
CommNet Sukhbaatar et al. (2016)	✓	✗	✗	✗	Explicit (Message Passing)	Discrete
QMIX Rashid et al. (2018)	✓	✗	✗	✗	None	Discrete
MADDPG Lowe et al. (2017)	✓	✗	✗	✗	None	Discrete, Continuous
RMAPPO Yu et al. (2022)	✓	✓ *	✗	✗	None	Discrete
MAGIC Niu et al. (2021)	✓	✗	✗	✗	Explicit (Message Passing)	Discrete
ROLA Xiao et al. (2021)	✓	✓	✗	✗	None	Discrete
MACRPO (Ours)	✓	✓	✓	✓	None	Discrete, Continuous

*Note: While RMAPPO, uses recurrent layers, it does not utilize meta-trajectories or employ our proposed advantage estimation with cooperation control.

## 3 Background

### 3.1 Markov games

In this work, we consider a multi-agent extension of Partially Observable Markov Decision Processes (MPOMDPs) (Gmytrasiewicz and Doshi, 2005), also called partially observable Markov games (Littman, 1994). It can also be modeled as Partially Observable Stochastic Games (POSGs) (Hansen et al., 2004). A Markov game for *N* agents is defined by a set of states S describing the possible configurations of all agents, a set of actions $U_{1},\ldots ,U_{N}$ and a set of observations $O_{1},\ldots ,O_{N}$ for each agent. The probability distribution of the next state as a function of the current state and actions is determined by a Markovian transition function $T:S\times U_{1}\times \cdots \times U_{N}\to S$ . Each agent *i* uses a stochastic policy $\pi_{\theta_{i}}:O_{i}\times U_{i}\to \left[0,1\right]$ , parametrized by $\theta_{i}$ , to choose an action. Upon the state transition, the agent receives a scalar reward $r_{i}:S\times U_{i}\to R$ . We consider games where the total reward can be decomposed to individual agent rewards $r_{i}$ . Each agent *i* aims to maximize the rewards for all agents in a cooperative way (Lowe et al., 2017).

### 3.2 Proximal policy optimization

Proximal Policy Optimization (PPO) is a family of policy gradient methods for solving reinforcement learning problems, which alternate between sampling data through interaction with the environment and optimizing a surrogate objective function using stochastic gradient descent while limiting the deviation from the policy used to collect the data (Schulman et al., 2017). PPO aims to maximize the clipped expected improvement of the policy (Equation 1).

$$L^{\mathrm{CLIP}}\left(\theta \right)={\hat{E}}_{t}\left[min\left(f_{t}\left(\theta \right){\hat{A}}_{t},clip\left(f_{t}\left(\theta \right),1-\epsilon ,1+\epsilon \right){\hat{A}}_{t}\right)\right]$$ (1)

where ${\hat{A}}_{t}$ is the advantage obtained by Generalized Advantage Estimation (GAE), ϵ is a hyperparameter, and $f_{t}\left(\theta \right)$ denotes the probability ratio $f_{t}\left(\theta \right)\equiv \frac{\pi_{\theta }\left(u_{t}|o_{t}\right)}{\pi_{\theta_{\mathrm{old}}}\left(u_{t}|o_{t}\right)}$ for importance sampling. The clipping prevents excessively large policy updates.

In addition to the expected improvement, the total objective function for PPO incorporates a loss function for a critic network required for GAE and an entropy bonus term to encourage exploration, resulting in the total objective (Equation 2) (Schulman et al., 2017).

$$L_{t}^{\mathrm{CLIP+V F+S}}\left(\theta \right)={\hat{E}}_{t}\left[L_{t}^{\mathrm{CLIP}}\left(\theta \right)-c_{1}L_{t}^{VF}\left(\theta \right)+c_{2}S\left[\pi_{\theta }\right]\left(o_{t}\right)\right]$$ (2)

where $c_{1}$ , $c_{2}$ are weight factors, S denotes the entropy bonus, and $L_{t}^{VF}$ is a squared-error loss for the critic

$$L_{t}^{VF}\left(\theta \right)={\left(V_{\theta }\left(o_{t}\right)-V_{t}^{\mathrm{targ}}\right)}^{2}$$ (3)

In the above equations, $V_{\theta }\left(o_{t}\right)$ is the state-value function and θ denotes the combined parameter vector of actor and critic networks. PPO uses multiple epochs of minibatch updates for each set of sampled interactions.

## 4 Methods

In this section, we introduce the problem setting and provide an overview of our proposed solution. We then describe the two key components of our approach in detail: (1) a centralized critic based on a recurrent neural network (RNN), which utilizes a novel meta-trajectory to capture inter-agent dynamics, and (2) an advantage estimation technique that incorporates weighted rewards, controlled by a parameter to adjust the level of cooperation between agents. Finally, we present a summary of the proposed MACRPO algorithm.

For clarity, a detailed description of all symbols and variables used in the equations throughout the manuscript is provided in the Nomenclature (Supplementary Appendix A1).

### 4.1 Problem setting and solution overview

Multi-agent systems operating in partially observable environments face significant challenges due to limited information and the absence of direct communication between agents. These factors hinder agents’ ability to coordinate effectively and learn optimal policies, which can lead to suboptimal performance in cooperative tasks. Effective information sharing among agents is critical for improving performance and accelerating learning in multi-agent reinforcement learning (MARL) (Gupta et al., 2017; Foerster et al., 2018; Terry et al., 2020). In this work, we aim to enhance information sharing in multi-agent environments, going beyond the traditional approach of merely sharing parameters across actor networks.

We introduce the Multi-Agent Cooperative Recurrent Proximal Policy Optimization (MACRPO) algorithm, a cooperative MARL approach based on the centralized training and decentralized execution (CTDE) paradigm. MACRPO addresses the partial observability and lack of direct communication by integrating two novel mechanisms that significantly improve information sharing and cooperation between agents:

• **Recurrent Critic Architecture**: The critic network leverages a recurrent neural network (RNN) trained on a meta-trajectory, which is constructed by combining the trajectories collected from all agents (detailed in Section 4.2). This allows the critic to model the interactions and dependencies between agents over time, capturing both agent-specific and collective behavior effectively.

• **Advantage Function Estimator**: A novel advantage function estimation approach that combines the individual agents’ rewards and value functions. This estimator incorporates a control parameter to dynamically adjust the level of cooperation between agents, enabling MACRPO to flexibly handle different cooperation strategies (explained in Section 4.3).

By combining these two components, MACRPO enables more effective cooperation and improves policy learning in complex, partially observable multi-agent environments, addressing the core challenges of coordination, partial observability, and dynamic cooperation.

### 4.2 MACRPO framework

The proposed MACRPO framework consists of one recurrent actor, similar to Foerster et al. (2018), and one recurrent critic network, as illustrated in Figure 1. To consider the partial observability of multi-agent settings, we use recurrent LSTM layers in both actor and critic networks to allow the integration of information over time.

The actor-network architecture is composed of a stack of Embedding, LSTM, and Linear layers and is trained using trajectories collected by all agents. We denote the shared weights of actors with $\theta_{a}$ and use the same, latest weights for all agents. The behaviors of different agents vary because of stochasticity and differences in their inputs. Denoting the trajectory data for episode k with length *T* for agent *i* as

$$\tau_{i}^{k}=\left(o_{1}^{i},u_{1}^{i},r_{1}^{i},\ldots ,o_{T}^{i},u_{T}^{i},r_{T}^{i}\right),$$

where o , u , and r represent the observations, actions, and rewards of the agents, respectively. The training data for the actor is then $D_{A}=\left(\tau_{1}^{1},\ldots ,\tau_{i}^{k},\ldots \right)$ .

To enable the critic network to integrate information across both agents and time, we introduce a **meta-trajectory**, which concatenates the trajectories of all agents during each roll-out. The critic network, consisting of Embedding, LSTM, and Linear layers, is trained on this meta-trajectory, allowing it to capture the interactions between agents and the temporal dynamics of the environment (see Figure 1).

To ensure that the critic network does not develop a dependency on a specific ordering of agents, we randomize the order of agents each time we generate a meta-trajectory. Specifically, for each meta-trajectory, we fix a random order of agents throughout the trajectory’s time steps, ensuring consistency within the trajectory. However, a different random order of agents is chosen for each new meta-trajectory. This randomization prevents the critic from associating certain positional patterns with specific agents, thereby reducing any positional bias in the learned policy. By varying the order of agents in each meta-trajectory, the critic is encouraged to focus on the agents’ observations, actions, and rewards independently of their positional index, resulting in a more generalized and robust policy that is less sensitive to the order in which agents are presented.

The training data for the critic network is structured similarly to the actor’s training data, but with the meta-trajectory as input. Let the meta-trajectory for episode k of length T for N agents be represented as:

$$\begin{array}{l} \mu^{k}=\left(o_{1}^{1},\ldots ,o_{1}^{N},u_{1}^{1},\ldots ,u_{1}^{N},r_{1}^{1},\ldots ,r_{1}^{N},\ldots \right.\left.,o_{T}^{1},\ldots ,o_{T}^{N},u_{T}^{1},\ldots ,u_{T}^{N},r_{T}^{1},\ldots ,r_{T}^{N}\right) \end{array}$$

The complete training data for the critic is then $D_{C}=\left(\mu^{1},\ldots ,\mu^{k},\ldots \right)$ .

By leveraging the above meta-trajectory, the critic network receives information from all agents to capture the agents’ history, the interactions between them, and the environment dynamics, all captured by the hidden state. In other words, MACRPO is able to consider temporal dynamics using the LSTM layer, which incorporates a history of states and actions across all agents. Modeling temporal dynamics allows the latent space to model differential quantities such as the rate of change (derivative) between the distance of two agents and integral quantities such as the running average of the distance.

**FIGURE 3:**
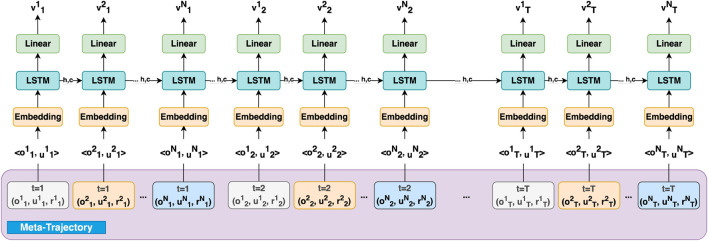
Centralized Critic Network Architecture, which utilizes the created meta-trajectory composed of the observations, actions, and rewards from all agents over multiple time-steps. The meta-trajectory allows the critic to evaluate the value function $\left(v_{i}^{t}\right)$ by considering the joint experiences of all agents, capturing the interactions and dependencies between them. The centralized critic is used during training to improve cooperation between agents, while the actor networks operate independently during execution. Note that u , v , o , and r denote action, value, observation, and reward respectively, and the superscripts and subscripts represent the agent number and time-step.

Additionally, the hidden state of recurrent networks can be viewed as a communication channel that allows information to flow between agents to create richer training signals for actors during training. The network will update the hidden state in each time step by getting the previous hidden state and the data from the agent *i* in that time step. The network architectures for actor and critic are shown in Figures 2 and 3. It is important to note that the critic network is only needed during training and that the optimized policy can be deployed using only the actor such that the agents are able to operate in a fully distributed manner without communication.

### 4.3 Objective function

In addition to the LSTM layer, we propose a novel advantage function estimator based on weighted discounted returns using a parameter that controls the agents’ cooperation level and integrates information across agents. We consider the $V_{t}^{\mathrm{targ}}$ in Equation 3 as discounted return and propose to calculate it for agent *i* at time *t* as

Algorithm 1 MACRPO.

1: Randomly initialize actor and critic networks’parameters $\theta_{c}$ and $\theta_{a}$

2: **for** iteration = 1, 2, … **do**
3:   **for** environment = 1, 2, …, E **do**
4:     Run all N agents with the latest trainedweights in the environment for T time stepsand collect data
5:     Combine collected trajectories by allagents according to Figure 1
6:     Compute discounted returns and advantageestimates using Equations 4, 6
7:   **end for**
8:   **for** epoch = 1, …, K **do**
9:     **for** minibatch = 1, … , M **do**
10:      Calculate the loss functions usingEquations 8, 9
11:      Update Actor and Critic parametersvia Adam
12:     **end for**
13:  **end for**
14: **end for**

$$R_{t}^{i}={\bar{r}}_{t}+\gamma {\bar{r}}_{t+1}+\cdots +\gamma^{T-t+1}\bar{V}\left(o_{T}^{i}\right)$$ (4)

where

$${\bar{r}}_{t}=\frac{r_{t}^{i}+\beta \underset{j\neq i}{\sum }r_{t}^{j}}{N},\;\bar{V}\left(o_{T}^{i}\right)=\frac{V\left(o_{T}^{i}\right)+\beta \underset{j\neq i}{\sum }V\left(o_{T}^{j}\right)}{N}$$ (5)

where $r_{t}^{i}$ is the reward for agent *i* at time *t*, γ is the discount factor, β is the cooperation control parameter used for rewards of other agents, and $V\left(o_{T}^{i}\right)$ is the value for the final state of agent *i*. The advantage for each agent *i* is then calculated as

$${\hat{A}}_{t}^{i}=\delta_{t}^{i}+\left(\gamma \lambda \right)\delta_{t+1}^{i}+\cdots +{\left(\gamma \lambda \right)}^{T-t+1}\delta_{T-1}^{i}$$ (6)

where

$$\delta_{t}^{i}=\frac{1}{N}\left[r_{t}^{i}+\gamma V\left(o_{t+1}^{i}\right)-V\left(o_{t}^{i}\right)+\beta \underset{j\neq i}{\sum }\left(r_{t}^{j}+\gamma V\left(o_{t+1}^{j}\right)-V\left(o_{t}^{j}\right)\right)\right]$$ (7)

where λ is the temporal difference factor of the GAE algorithm, and $V\left(o_{t}^{i}\right)$ is the state-value at time *t* for agent *i*.

The intuition behind the weighting is that each agent’s rewards are likely to be affected most by its own action choice but that the actions taken by other agents can also affect the reward. In addition, the β parameter can be interpreted as a control parameter for the cooperation level between agents, which is manually tuned based on the cooperative nature of the task. This heuristic is related to credit assignment between agents and provides a trade-off between optimizing the policy considering only individual rewards ( β=0 and no cooperation between agents), which could lead to sub-optimal total reward when individual rewards are in conflict with each other, and optimizing the policy using the sum of all rewards ( β=1 and full cooperation between agents), which could lead to challenging assignment of credit between agents. One should note that policy optimization is performed across all agents such that in the end, the expected rewards over all agents are maximized, independent of the choice of β .

MACRPO uses separate networks for actors and critics. Therefore, the objective functions of the actor and critic networks are separate, in contrast to PPO. The actor’s objective function in the case of the shared weight is defined as

$$L_{t}^{\mathrm{CLIP+S}}\left(\theta_{a}\right)={\hat{E}}_{t}\left[L_{t}^{\mathrm{CLIP}}\left(\theta_{a}\right)+cS\left[\pi_{\theta_{a}}\right]\left(o_{t}\right)\right]$$ (8)

and the critic’s objective function is

$$L_{t}^{VF}\left(\theta \right)={\left(V_{\theta_{c}}\left(o_{t}\right)-V_{t}^{\mathrm{targ}}\right)}^{2}$$ (9)

where $\theta_{c}$ are the parameters of the critic A parallelized version of the MACRPO algorithm is shown in Algorithm 1.

## 5 Experiments

This section presents empirical results to evaluate the performance of our proposed method, MACRPO. We provide a comprehensive evaluation by testing MACRPO across three diverse and well-established multi-agent environments: DeepDrive-Zero (Quiter, 2020), Multi-Walker (Gupta et al., 2017), and Particle (Mordatch and Abbeel, 2018). These environments were selected to represent a range of cooperative multi-agent tasks, differing in terms of action spaces (continuous and discrete), task dynamics, and cooperation requirements, ensuring a thorough assessment of MACRPO’s generalizability.

In addition to evaluating MACRPO, we compare its performance against several state-of-the-art (SOTA) algorithms, including MADDPG (Lowe et al., 2017), RMAPPO (Yu et al., 2022), and QMIX (Rashid et al., 2018), which are widely regarded as benchmarks in cooperative multi-agent reinforcement learning. The results demonstrate the effectiveness of MACRPO in addressing the challenges posed by these diverse environments. Furthermore, we conduct ablation studies to analyze the impact of each component of our approach, focusing on the meta-trajectory and cooperative advantage function, which are key to improving coordination between agents.

Together, these experiments provide a robust evaluation of MACRPO’s capabilities, demonstrating its adaptability and effectiveness across different settings without the need for additional static experiments. The comprehensive set of results, combined with the comparisons to SOTA methods, illustrates the advantages of our approach in multi-agent reinforcement learning.

### 5.1 Test environments

We evaluate MACRPO in three benchmark multi-agent environments: DeepDrive-Zero (Quiter, 2020), Multi-Walker (Gupta et al., 2017), and Particle (Mordatch and Abbeel, 2018). These environments were chosen for their diversity in task dynamics, action spaces (continuous and discrete), and cooperation requirements. They provide a comprehensive evaluation of our method’s generalizability across different multi-agent settings..

**FIGURE 4:**
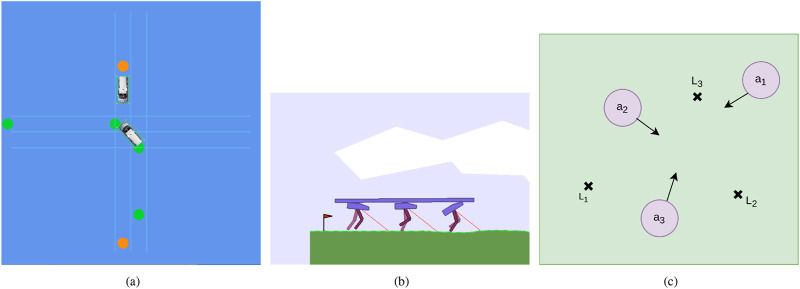
Considered MARL simulation environments **(A)** DeepDrive-Zero environment: an unprotected left turn scenario, **(B)** Multi-Walker environment, **(C)** Particle environment: cooperative navigation.

• **DeepDrive-Zero Environment:** Several autonomous driving simulators can be used for multi-agent simulation (Dosovitskiy et al., 2017; Santara et al., 2021; Quiter, 2020). In this work, we use DeepDrive-Zero (Quiter, 2020), because we do not need to deal with image data and also need a fast simulation environment for training. DeepDrive-Zero is a very fast and 2D simulation environment for self-driving cars that uses a bike model for the cars. We use the unsignalized intersection scenario in this work, which is shown in Figure 4A. To test our algorithm, we consider two cars in the environment, one starts from the south and wants to follow the green waypoints to do an unprotected left-turn, and the other one starts from the north and wants to go to the south and follow the orange waypoints. The agents need to learn to cooperate and negotiate to reach their destination without any collision.

• **Multi-Walker Environment:** The multi-walker environment is a multi-agent continuous control locomotion task introduced in Gupta et al. (2017). The environment contains agents (bipedal walkers) that can actuate the joints in each of their legs and convey objects on top of them. Figure 4B shows a snapshot from the environment.

• **Cooperative Navigation in Particle Environment:** Using the particle environment package from OpenAI (Lowe et al., 2017), we created a new environment based on the cooperative navigation environment. This new environment consists of N agents and N landmarks, and agents must avoid collisions and cooperate to reach and cover all landmarks. Figure 4C shows the simulation environment.

Each environment presents unique challenges related to agent cooperation, partial observability, and decentralized decision-making, allowing for a thorough evaluation of MACRPO’s performance. Check Supplementary Appendix A2 for more details about the environments.

### 5.2 Ablation study

Four ablations were designed to evaluate each novelty. The name of the method and the explanation shows which ablation has Feed-forward or LSTM or how information is shared in that ablation. In all cases, the parameter sharing proposed in Gupta et al. (2017) and Terry et al. (2020) was used:

• **FF-NIC:** (*Feed-forward multi-layer perceptron (MLP) network + no information combination*): two feed-forward neural networks for actor and critic. The GAE is calculated using the single-agent PPO GAE equation (Schulman et al., 2017). There is no LSTM layer or reward and value functions combination for information sharing in this case.

• **FF-ICA:** (*Feed-forward MLP network + information combination using the advantage estimation function*): This case is similar to the previous case, but the GAE is calculated using Equation 6 to show the effect of mixing reward and value functions for information sharing. There is no LSTM layer in this case too.

• **LSTM-NIC:** (*LSTM network + no information combination*): two networks with LSTM layers for actor and critic. There is no information sharing between agents through GAE calculation or the LSTM’s hidden state. The GAE is calculated using the single-agent PPO GAE equation (Schulman et al., 2017).

• **LSTM-ICA:** (*LSTM network + information combination using the advantage estimation function but not through the LSTM layer*): This case is identical to the previous case, but the GAE is calculated using Equation 6.

• **LSTM-ICF:** *(LSTM network + information sharing using both the advantage estimation function and an LSTM layer in the critic network (full proposed method))*: two networks with LSTM layers for actor and critic. In addition to parameter sharing between actors, the information integration is done through both the advantage estimation function and the LSTM’s hidden state in the centralized critic network, shown in Figure 1.

Also, in order to see the effect of the β value in Equation 5 and Equation 7, the proposed method was evaluated with different β values which shows different cooperation levels between agents.

All experiments were repeated with identical random seeds for each method to reduce the effect of randomness. Hyperparameters used in MACRPO for three environments are detailed in Supplementary Appendix A4.

#### 5.2.1 DeepDrive-Zero environment

**FIGURE 5:**
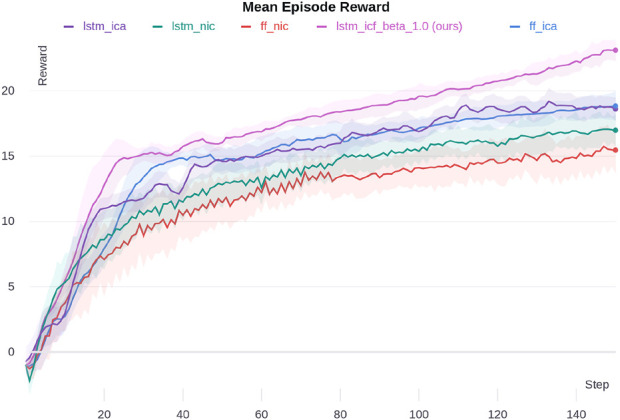
Mean episode reward for different ablations in the DeepDrive-Zero environment. The shaded area shows one standard deviation.

We ran all ablations for ten random seeds in the DeepDrive-Zero environment to test our proposed method. We used self-play in simulations and used the latest set of parameters for actors in each episode. The results are shown in Figure 5. The *x*-axis shows the number of training iterations. In each iteration, we ran 100 parallel environments for 3,000 steps and collected data. Next, we updated actors and critic networks using the collected data. After each iteration, we ran the agents for 100 episodes, took the mean of these episodes’ rewards (the sum of all agents’ rewards), and plotted them. The shaded area shows one standard deviation of episode rewards. The hyperparameters used in the MACRPO algorithm are listed inSupplementary Table 2 (Supplementary Appendix A4).

The proposed algorithm, LSTM-ICF, outperforms the ablations. The next best performances are for LSTM-ICA and FF-ICA, which are almost the same. Moreover, information integration in the advantage function, in both FF-ICA and LSTM-ICA, improves the performance compared to FF-NIC and LSTM-NIC; however, the achieved performance gain in the fully connected case is higher. The FF-ICA surpasses LSTM-NIC, which shows the effectiveness of sharing information across agents through the proposed advantage function, even without an LSTM layer. The addition of the LSTM layer in LSTM-ICF further enhances performance by capturing temporal dependencies between agents’ actions and observations, which are crucial in dynamic multi-agent environments like DeepDrive Zero. The LSTM enables the critic to remember past interactions and predict future dependencies, which provides a more holistic understanding of agent dynamics over time. This temporal information allows for more informed decision-making and improves the ability of agents to anticipate and respond to each other’s actions effectively. Consequently, the use of LSTM leads to a more coordinated and adaptive behavior across agents, which is reflected in the superior performance of LSTM-ICF compared to other ablations.

**FIGURE 6:**
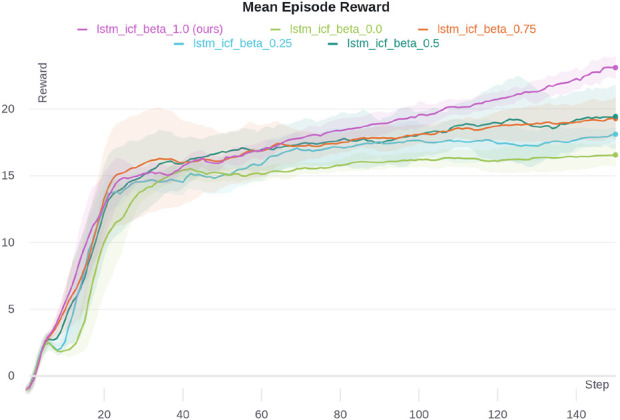
Mean episode reward for different β values in the DeepDrive-Zero environment. The shaded area shows one standard deviation.

Figure 6 shows the analysis of the effect of different β values in Equation 4, Equation 5, and Equation 7. The best performance is achieved with β=1 , which corresponds to full cooperation between agents. In the DeepDrive Zero environment, full cooperation enables agents to make decisions that maximize collective rewards, which is particularly advantageous in tasks that require closely coordinated actions to avoid collisions and optimize traffic flow.

As β decreases, the level of cooperation between agents is reduced, leading to a decline in performance. Lower values of β (closer to 0) encourage more independent behavior, which can result in suboptimal coordination in this environment, as agents prioritize their own rewards over group performance. This trend illustrates the importance of cooperation in achieving optimal outcomes in tightly coupled tasks, where synchronized actions among agents are critical.

The β parameter provides MACRPO with flexibility to adapt to different task requirements by adjusting the degree of cooperation. While higher β values are suitable for tasks requiring full cooperation, lower β values may be advantageous in settings where agents have individual objectives but can benefit from limited coordination. While we demonstrate the effect of different β values in this environment, results for other environments are provided only for β∈{0,1} .

To achieve smooth driving performance, a curriculum-based learning method and a gradual weight increase of reward factors were used. The weights of Jerk, G-force, steering angle change, acceleration change, and going out of the lane in the reward function were gradually increased to $3.3\times 10^{-6}$ , 0.1, 3, 0.05, and 0.3, respectively. We then added termination of episodes for lane violations to force cars to stay between the lanes. After curriculum learning and smoothing the driving behavior, the cars follow the waypoints to reach their destination. The car that starts from the bottom and wants to make a left turn yields nicely for the other agent if they reach the intersection simultaneously and then make the left turn, and if it has time to cross the intersection before the other agent arrives, it does. A video of the final result can be found in the supplementary materials.

#### 5.2.2 Multi-walker environment

We ran 20 parallel environments and 2,500 time steps during each update iteration for the Multi-Walker environment. After each iteration, we ran agents for 100 episodes and plotted the mean of these episodes’ rewards. Each episode’s reward is the sum of all the agents’ rewards. Ten different random seeds are used for each ablation. We also used the latest set of parameters for all actors. The hyperparameters used in the MACRPO algorithm are listedSupplementary Table 2 (Supplementary Appendix A4).

**FIGURE 7:**
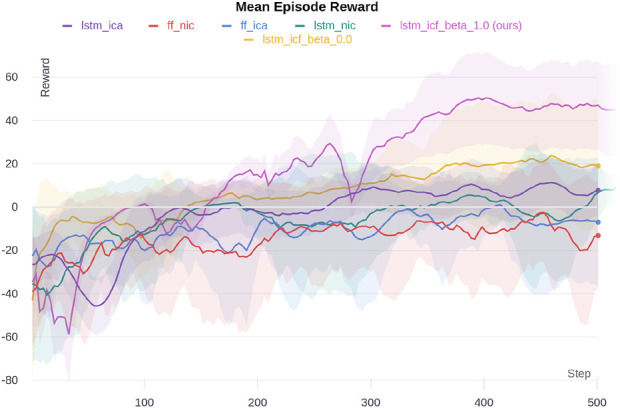
Multi-Walker simulation results for different ablations. The shaded area shows one standard deviation.

Figure 7 shows a massive performance improvement of our proposed method, LSTM-ICF with β=1 , when compared to ablations. LSTM-ICF with β=0 , which uses information integration through only the LSTM layer, has the next best performance. The LSTM layer enables the critic network to capture temporal dependencies between agents’ actions and states, which is crucial in this environment where agents need to coordinate to balance and move a shared object. By retaining information about past interactions, the LSTM facilitates coordinated behavior, allowing agents to anticipate each other’s movements effectively, which contributes to the superior performance of LSTM-based methods. After these two, LSTM-ICA, which performs information integration using the advantage estimation function, performs better than the FF-ICA, FF-NIC, and LSTM-NIC cases.

The effect of the β value and information sharing through the advantage estimation function can be seen in the performance improvement from LSTM-ICF with β=0 to LSTM-ICF with β=1 and from FF-NIC to FF-ICA. In the Multi-Walker environment, where agents must coordinate to move a shared object without dropping it, the cooperation level controlled by β plays a critical role. A higher β value (closer to 1) enables agents to prioritize collective rewards, fostering synchronized movement and reducing the likelihood of dropping the object. This emphasis on group performance is reflected in the superior results for LSTM-ICF with β=1 , where full cooperation between agents leads to optimal performance. In contrast, lower β values (closer to 0) encourage agents to act more independently, which may result in less effective coordination and increased instability in this task. The improvement from FF-ICA to LSTM-ICF further demonstrates the impact of integrating temporal dependencies via the LSTM layer, which is essential in environments like Multi-Walker that require sustained coordination over time. The β value in FF-ICA is set to 1, indicating that it also benefits from full cooperation, but lacks the temporal integration capabilities provided by the LSTM. A video of the trained model can be found in the supplementary materials.

#### 5.2.3 Cooperative navigation in particle environment

In the particle environment, in each iteration, we ran 20 parallel environments to collect data for 2,500 time steps and used that data to update the network. The agents were then evaluated using the trained weights for 100 episodes. We ran the simulation with six random seeds. MACRPO hyperparameters are shown in Supplementary Table 2 (Supplementary Appendix A4).

**FIGURE 8:**
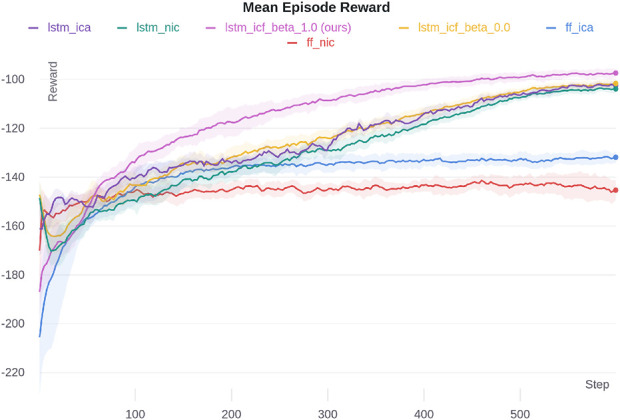
Particle environment simulation results for different ablations. The shaded area shows one standard deviation.

The results of this environment are depicted in Figure 8. Similar to the other two environments, the proposed LSTM-ICF with β=1 outperforms all ablations. This environment requires agents to coordinate their navigation to avoid collisions, making temporal awareness of others’ trajectories essential. The LSTM layer significantly enhances performance by allowing agents to capture these temporal dependencies, leading to smoother coordination and reduced collision rates. This capability is particularly beneficial in environments with continuous movement and high inter-agent interaction.

The effect of the β value is also apparent in the performance differences between LSTM-ICF with β=1 and LSTM-ICF with β=0 . Higher cooperation (achieved with β=1 ) enables agents to prioritize group navigation goals, effectively balancing collision avoidance and path optimization. Lower cooperation levels, as seen with β=0 , encourage more independent agent behavior, which can lead to suboptimal group coordination in this scenario. The next best performance is achieved with LSTM-ICF with β=0 , showing that even without full cooperation, the temporal integration from the LSTM layer provides a significant advantage. The LSTM-ICA’s performance is almost identical to LSTM-ICF with β=0 , indicating that both the LSTM layer and the advantage function contribute similarly to performance improvements, and that information sharing through these mechanisms provides notable benefits over the baseline LSTM-NIC.

The results of the ablation study clearly demonstrate the impact of both proposed enhancements—information integration through the advantage function estimation and temporal dependency capture via the LSTM layer—on improving performance. The findings confirm that these improvements are not spurious but are directly attributable to the proposed modifications.

In general, the cases with the LSTM layer consistently perform better than the feed-forward counterparts, even in the FF-ICA case, which integrates information solely through the advantage function. This highlights the importance of capturing temporal dependencies via the LSTM network for enhanced coordination and navigation in highly interactive environments.

Our study also explored the role of the cooperation control parameter β , where β=1 corresponds to full cooperation by optimizing the total reward across all agents, which is the primary objective in these environments. Although values of β less than one were considered as a potential way to mitigate the credit assignment problem, the experiments showed that β=1 consistently provided the best performance. This suggests that full cooperation is more beneficial in these fully cooperative settings, as lower values of β did not improve credit assignment enough to offset the loss in overall performance.

Additionally, while both proposed ideas yield performance improvements, their relative effectiveness varies across environments. In the DeepDrive-Zero environment, information integration through the advantage function has a slightly greater impact than the LSTM layer. In contrast, the LSTM layer is more effective in the Multi-Walker environment, while in the Particle environment, both enhancements contribute equally to performance gains.

In our study, the tested environments—DeepDrive Zero, Multi-Walker, and Particle—are fully cooperative multi-agent settings, where agents benefit from high β values that encourage full cooperation. These tightly coupled tasks require agents to work closely to achieve a common goal, making high cooperation levels ideal. However, MACRPO is adaptable and could be applied to partially cooperative environments, where agents have individual goals but can still benefit from limited coordination. In such scenarios, lower β values could allow agents to act more independently while maintaining a degree of cooperation, balancing individual and group objectives.

A video of the trained model can be found in the supplementary materials.

### 5.3 Comparison to state-of-the-art methods

We compared the proposed method with several state-of-the-art algorithms in each environment. Our method is compared against several single-agent baselines with shared parameters across agents (DQN, RDQN, A2C, DDPG, PPO, SAC, TD3, APEX-DQN, APEX-DDPG, and IMPALA), which were tested in Terry et al. (2020). We also compared our method to state-of-the-art multi-agent approaches such as MAGIC, (Niu et al., 2021), IC3Net (Singh et al., 2019), GA-Comm (Liu et al., 2020), CommNet (Sukhbaatar et al., 2016), MADDPG (Lowe et al., 2017), RMAPPO (Yu et al., 2022), and QMIX (Rashid et al., 2018).

The architecture and hyperparameters used for PPO, IMPALA, A2C, SAC, APEX-DQN, Rainbow-DQN, DQN, APEX-DDPG, DDPG, TD3, and QMIX are taken from Terry et al. (2020) which has an open source implementation 1. For MADDPG (Lowe et al., 2017), we used the original source code 2, for RMAPPO (Yu et al., 2022) we used their open source code 3, and for MAGIC, IC3Net, CommNet, and GA-Comm we used the open source implementation 4. We performed hyperparameter tuning using grid search to optimize performance for each method.

Note that some of the official implementations of baselines we used here do not support both discrete and continuous action spaces and we did not modify the code. The non-reported results for some baselines in the paper’s tables and charts are due to this. In addition, we tried to use the discretized version of the DeepDrive-Zero environment for algorithms with discrete action space which may cause poor performance.

Each agent’s goal in MACRPO is to maximize the total reward of all agents, while the goal of other methods is to maximize the total reward of each agent without considering other agents’ rewards in their objective function. In order to have a more fair comparison, We report the result for our method when β=0 too. The results are shown in Table 2. The table contains some empty fields due to the fact that some algorithms do not support continuous or discrete action spaces. Check Supplementary Appendix A3 for more details.

**TABLE 2:** Comparing performance of our method with state-of-the-art approaches. Numbers show the average reward in each environment for ten random seeds, except for the Multi-Walker environment which is 1,000 random seeds.

Method	DeepDrive-Zero	Multi-Walker	Particle
DQN	4	−100000	−151.8
RDQN	6	−100000	153.2
A2C	0.5	−27.6	−148.6
DDPG	2	−57.8	—
PPO	16	41	−144.3
SAC	−1.5	−16.9	−143.7
TD3	−1	−8	—
APEX-DQN	8	−100000	−136.2
APEX-DDPG	14	−23	—
IMPALA	−0.66	−88	−155.2
MADDPG	−0.1	−96	−98.3
QMIX	−0.9	−24	−155.6
MAGIC	3.1	—	−114
IC3Net	2.1	—	−117
GA-Comm	1.9	—	−119
CommNet	1.6	—	−115
RMAPPO	17.06	—	−131
Ours (β=0)	17.3	24.2	−100.7
Ours (full model)	23.7	47.8	−95.8

#### 5.3.1 DeepDrive-Zero environment

In this environment, our full method and also the case with β=0 achieved the highest average reward. The next best was RMAPPO which performed close to our method in the case of β=0 and then PPO with parameter sharing between agents followed by APEX-DQN and APEX-DDPG. A version of the environment with discretized action space was used for algorithms with discretized action space.

#### 5.3.2 Multi-walker environment

Similar to the previous environment, the proposed method outperformed other methods by a large margin with an average reward of 47.8. Next, PPO with parameter sharing had the second-best performance with a maximum average reward of 41. Our method with β=0 achieved the third-best average reward. Some algorithms, such as RMAPPO, CommNet, GA-Comm, IC3Net, and MAGIC, do not support continuous action spaces and are marked with a dash in the table.

#### 5.3.3 Cooperative navigation in particle environment

As in both previous environments, our approach outperformed other approaches in this environment as well, although the difference was minor compared to MADDPG. Our method with β=0 is in the third place after MADDPG with a small margin. We used a categorical distribution instead of a multivariate Gaussian distribution in this environment with discrete action space. Algorithms with continuous action spaces were not tested in this environment, and are marked with a dash in the table. Adapting these algorithms for discrete action environments could be achieved using the same trick, but we did not change the standard implementation for baselines.

It is evident from the reported results that RMAPPO performance in the DeepDrive-Zero environment is satisfactory and comparable to our method in the case of β=0 and that it is average in the Particle environment. As the current implementation of RMAPPO does not support continuous action spaces, we could not test this method in the Multi-Walker environment. Additionally, we conducted hyperparameter searches for RMAPPO, but since this method aims to recommend a set of modifications and hyperparameters that will improve PPO’s performance for multi-agent systems, we did not deviate too far from the main hyperparameters. The performance of MADDPG is not also good in DeepDerive-Zero and Multi-Walker environments. However, it performs well when used in the Particle environment.

All hyperparameters for each algorithm are included in Supplementary Appendix A4.

The results show that the performance benefit given by the two proposed ways of sharing information across agents is significant such that the method outperforms state-of-the-art algorithms.

## 6 Conclusion and future work

In this paper, MACRPO, a centralized training and decentralized execution framework for multi-agent cooperative settings was presented. The framework is applicable to both discrete and continuous action spaces. In addition to parameter sharing across agents, this framework integrates information across agents and time in two novel ways: network architecture and the advantage estimation function. An ablation study in three environments revealed that both ways of information sharing are beneficial. Furthermore, the method was compared to state-of-the-art multi-agent algorithms such as MAGIC, IC3Net, CommNet, GA-Comm, QMIX, and MADDPG, as well as single-agent algorithms that share parameters between agents, such as IMPALA and APEX. The results showed that the proposed algorithm performed significantly better than state-of-the-art algorithms.

While MACRPO demonstrates strong performance in a range of tasks, there are some limitations. In environments with high-dimensional discrete action spaces, performance may be impacted due to the increased complexity of managing agent interactions. Additionally, MACRPO’s centralized critic, which processes a meta-trajectory of all agents, may face scalability challenges as the number of agents grows. For larger agent populations, integrating an attention mechanism could be a potential solution, allowing agents to selectively focus on critical information from others without processing data from all agents simultaneously. This enhancement could improve MACRPO’s efficiency and scalability in large-scale multi-agent environments.

Despite these limitations, MACRPO’s adaptable cooperation control parameter, β , makes it highly flexible for diverse multi-agent tasks, such as autonomous driving and collaborative robotics, where varying levels of cooperation are essential. Future work could further explore adaptive cooperation strategies and attention-based architectures to enhance MACRPO’s application scope and performance in complex, large-scale multi-agent systems.

## Data Availability

The datasets presented in this study can be found in online repositories. The names of the repository/repositories and accession number(s) can be found in the article/Supplementary Material.
