# Supplementary material for: MACRPO: Multi-agent cooperative recurrent policy optimization
Source: Front Robot AI. 2024 Dec 20;11:1394209. doi: 10.3389/frobt.2024.1394209 (PMC11695781; doi:10.3389/frobt.2024.1394209)
Supplement: Supplementary file 2 [file DataSheet1.pdf]

1 NOMENCLATURE

This section defines the symbols and variables used throughout the manuscript.

Table 1. List of symbols and variables used in the manuscript.

Symbol	Definition
o_i^t	Observation of agent i at time-step t.
u_i^t	Action taken by agent i at time-step t.
r_i^t	Reward received by agent i at time-step t.
v_i^t	Value function for agent i at time-step t.
π_i	Policy of agent i.
β	Cooperation control parameter for adjusting the level of cooperation between agents.
μ^k	Meta-trajectory for episode k, combining observations, actions, and rewards from all agents.
T	Length of an episode.
N	Number of agents in the environment.
D_C	Training dataset for the critic network, containing meta-trajectories.
δ_i^t	Temporal difference (TD) error for agent i at time-step t.
R_i	Discounted return for agent i.
A_i	Advantage function for agent i.

2 DETAILED ENVIRONMENT DESCRIPTIONS

2.1 DeepDrive-Zero Environment:

The observation space is a vector with continuous values. Each agent in the environment receives some information about itself, as well as information from other agents. This information can come from some modules like Perception, Localization, and HDMap in a self-driving car and be used by the decision-making and control modules. The observation vector for each agent contains some information about the agent itself like distance and angle to waypoints, velocity, acceleration, and distance to the left and right lanes, and also some information about the other agents like the relative velocity of the other agent to the ego agent, velocity and acceleration of the other car, angles to corners of the other agent, and distance to corners of the other agent.

Each action vector element is continuous from -1 to 1: steering, acceleration, and braking. Negative acceleration can be used to reverse the car, and the network outputs are scaled to reflect physically realistic values. This environment also has a discretized version that we used in discrete action methods.

The reward function is a weighted sum of several terms like speed, reaching the destination, collision, G-force, jerk, steering angle change, acceleration change, and staying in the lane. Initially, we used 0.5, 1, 4, 1×10^{-7}, 6×10^{-6}, 0.0001, 0.0001, 0.001 as weights, then used curriculum learning to smooth the driving behavior.

2.2 Multi-Walker Environment:

To keep the package balanced and move it as far to the right as possible, the walkers must coordinate their movements. A positive reward is given to each walker locally, based on the change in the package distance summed with 130 times the change in the walker's position. A walker is given a reward of -100 if

they fall, and all walkers receive a reward of -100 if the package falls while moving forward has a reward of 1. By default, the environment is done whenever a walker or package falls or when the walkers reach the edge of the terrain. The action space is continuous, with four values for torques applied to each walker's leg. The observation vector for each walker is a 32-dimensional vector that contains information about nearby walkers as well as data from some noisy LiDAR sensors.

2.3 Cooperative Navigation in Particle Environment:

We assign each agent a landmark and calculate its local reward based on its proximity to its landmark and collisions with other agents. As a result, agents will have different reward values; not one shared reward. Each agent's observation data is its position and velocity, as well as the relative position of other agents and landmarks. There are five discrete actions in the action space: up, down, left, right, and no move. After 25 time-steps, the episode ends.

3 COMPARISON TO STATE OF THE ART METHODS

To get a better idea of the performance of the state-of-the-art algorithms, the mean episode reward for different baseline algorithms in test environments is shown in Fig. 1, Fig. 2, and Fig. 3.

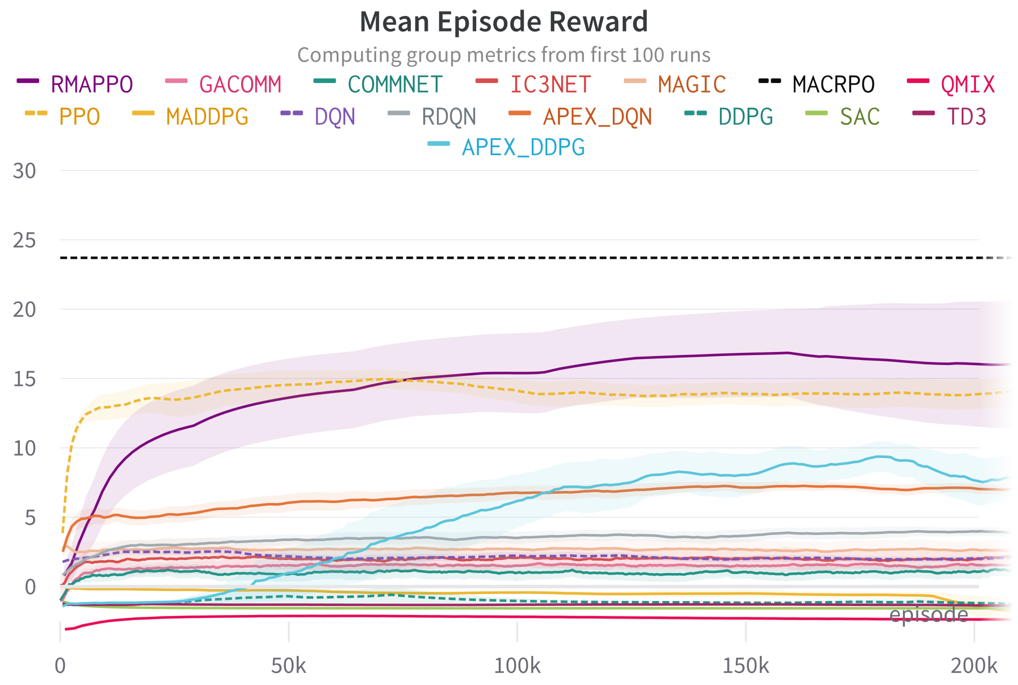

Figure 1. Analysis of baseline algorithms in the DeepDrive-Zero environment proposed in Terry et al. (2020). The shaded area shows one standard deviation.

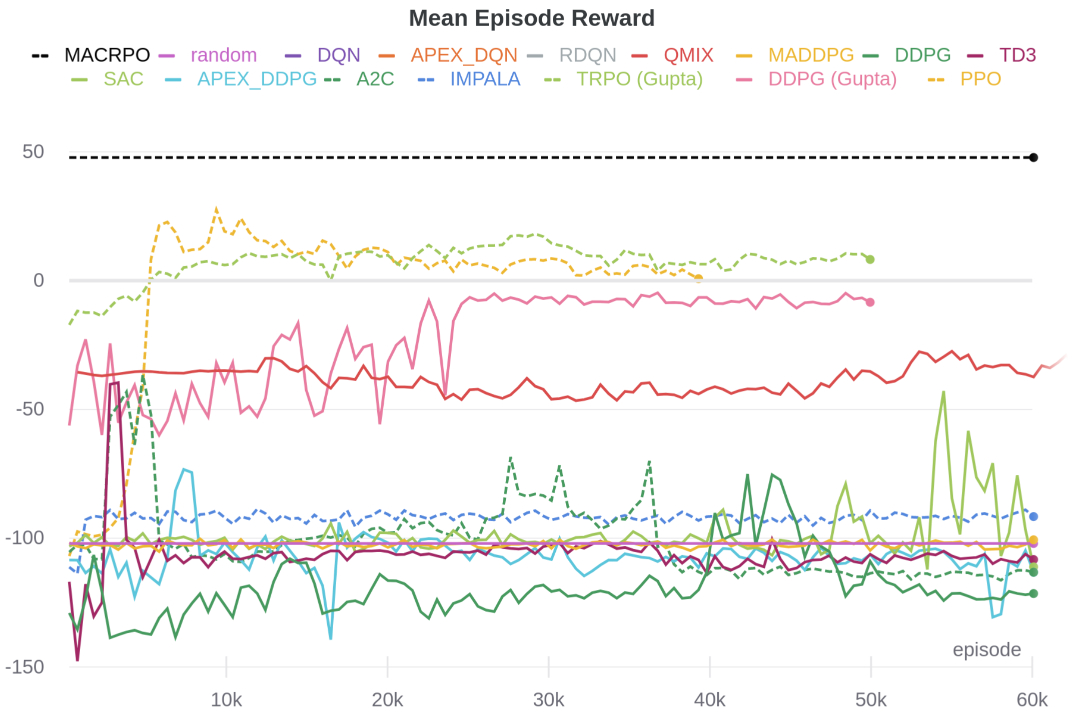

Figure 2. Analysis of baseline algorithms in the Multi-Walker environment proposed in Terry et al. (2020). The shaded area shows one standard deviation.

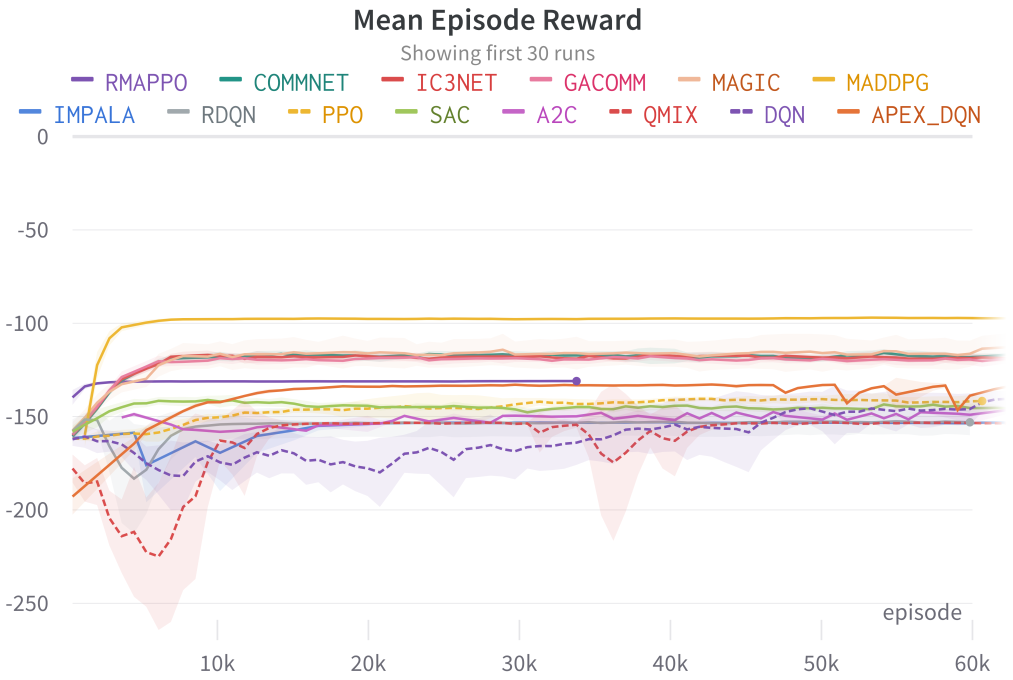

Figure 3. Analysis of baseline algorithms in the Particle environment proposed in Terry et al. (2020). The shaded area shows one standard deviation.

4 HYPERPARAMETERS

Hyperparameters used in MACRPO for three environments are described in Table 2.

Table 2. MACRPO hyperparameters for three MARL environments

Param.	DeepDrive-Zero	Multi-Walker	Particle
actor hidden size	64	32	128
critic hidden size	128	32	128
batch size	512	32	1500
discount	0.99	0.99	0.99
GAE lambda	0.94	0.95	0.95
PPO clip	0.15	0.3	0.2
PPO epochs	4	4	10
max grad norm	1.0	1.0	1.0
entropy factor	0.001	0.01	0.01
learning rate	0.0002	0.001	0.005
recurrent sequence length (time-step)	20	40	3
no. of recurrent layers	1	1	1

The architecture and hyperparameters used for other baselines are taken from Terry et al. (2020) with some fine-tuning to get better performance, and are shown in Tables 3, 4, and 5. Some hyperparameter values are constant across all RL methods for all environments. These constant values are reported in Table 6. We used the source code for all algorithms from Terry et al. (2020) except for MADDPG, which we used the original implementation (Lowe et al., 2017).

REFERENCES

Lowe, R., Wu, Y. I., Tamar, A., Harb, J., Abbeel, O. P., and Mordatch, I. (2017). Multi-agent actor-critic for mixed cooperative-competitive environments. In *Advances in neural information processing systems*. 6379–6390

Terry, J. K., Grammel, N., Hari, A., Santos, L., Black, B., and Manocha, D. (2020). Parameter sharing is surprisingly useful for multi-agent deep reinforcement learning. *arXiv preprint arXiv:2005.13625*

Table 3. Hyperparameters for three MARL environments

RL method	Hyperparameter	DeepDrive-Zero	Multi-Walker	Particle
PPO	sample_batch_size	100	100	100
	train_batch_size	5000	5000	5000
	sgd_minibatch_size	500	500	1000
	lambda	0.95	0.95	0.95
	kl_coeff	0.5	0.5	0.5
	entropy_coeff	0.01	0.01	0.001
	num_sgd_iter	10	10	50
	vf_clip_param	10.0	10.0	1.0
	clip_param	0.1	0.1	0.5
	vf_share_layers	True	True	True
	clip_rewards	True	True	False
	batch_mode	truncate_episodes	truncate_episodes	truncate_episodes
IMPALA	sample_batch_size	20	20	20
	train_batch_size	512	512	512
	lr_schedule	[[0, 5e-3], [2e7, 1e-12]]	[[0, 5e-3], [2e7, 1e-12]]	[[0, 5e-3], [2e7, 1e-12]]
	clip_rewards	True	True	False
A2C	sample_batch_size	20	20	20
	train_batch_size	512	512	512
	lr_schedule	[[0, 7e-3], [2e7, 1e-12]]	[[0, 7e-3], [2e7, 1e-12]]	[[0, 7e-3], [2e7, 1e-12]]
SAC	sample_batch_size	20	20	20
	train_batch_size	512	512	512
	Q_model	{activation: relu, layer_sizes: [266, 256]}	{activation: relu, layer_sizes: [266, 256]}	{activation: relu, layer_sizes: [266, 256]}
	optimization	{actor_lr: 0.0003, actor_lr: 0.0003, entropy_lr: 0.0003,}	{actor_lr: 0.0003, actor_lr: 0.0003, entropy_lr: 0.0003,}	{actor_lr: 0.0003, actor_lr: 0.0003, entropy_lr: 0.0003,}
	clip_actions	False	False	False
	exploration_enabled	True	True	True
	no_done_at_end	True	True	True
	normalize_actions	False	False	False
	prioritized_replay	False	False	False
	soft_horizon	False	False	False
	target_entropy	auto	auto	auto
	tau	0.005	0.005	0.005
	n_step	1	1	5
	evaluation_interval	1	1	1
	metrics_smoothing_episodes	5	5	5
	target_network_update_freq	1	1	1
	learning_starts	1000	1000	1000
	timesteps_per_iteration	1000	1000	1000
	buffer_size	100000	100000	100000

Table 4. Hyperparameters for DeepDrive-Zero, Multi-Walker, and Particle environments

RL method	Hyperparameter	DeepDrive-Zero	Multi-Walker	Particle
APEX-DQN	sample_batch_size	20	20	20
	train_batch_size	32	512	5000
	learning_starts	1000	1000	1000
	buffer_size	100000	100000	100000
	dueling	True	True	True
	double_q	True	True	True
Rainbow-DQN	sample_batch_size	20	20	20
	train_batch_size	32	512	1000
	learning_starts	1000	1000	1000
	buffer_size	100000	100000	100000
	n_step	2	2	2
	num_atoms	51	51	51
	v_min	0	0	0
	v_max	1500	1500	1500
	prioritized_replay	True	True	True
	dueling	True	True	True
	double_q	True	True	True
	parameter_noise	True	True	True
	batch_mode	complete_episodes	complete_episodes	complete_episodes
Plain DQN	sample_batch_size	20	20	20
	train_batch_size	32	512	5000
	learning_starts	1000	1000	1000
	buffer_size	100000	100000	100000
	dueling	False	False	False
	double_q	False	False	False
QMIX	buffer_size	10000	3000	100000
	gamma	0.99	0.99	0.99
	critic_lr	0.001	0.0005	0.001
	lr	0.001	0.0005	0.001
	grad_norm_clip	10	10	10
	optim_alpha	0.99	0.99	0.99
	optim_eps	0.00001	0.05	0.00001
	epsilon_finish	0.02	0.05	0.02
	epsilon_start	1.0	1.0	1.0
MADDPG	lr	0.001	0.0001	0.01
	batch_size	64	512	500
	num_envs	1	64	1
	num_cpus	1	8	1
	buffer_size	1e5	1e5	1e5
	steps_per_update	4	4	4

Table 5. Hyperparameters for DeepDrive-Zero and Multi-Walker

RL method	Hyperparameter	DeepDrive-Zero	Multi-Walker
APEX-DDPG	`sample_batch_size`	20	20
	`train_batch_size`	512	512
	`lr`	0.0001	0.0001
	`beta_annealing_fraction`	1.0	1.0
	`exploration_fraction`	0.1	0.1
	`final_prioritized_replay_beta`	1.0	1.0
	`n_step`	3	3
	`prioritized_replay_alpha`	0.5	0.5
	`learning_starts`	1000	1000
	`buffer_size`	100000	100000
	`target_network_update_freq`	50000	50000
	`timesteps_per_iteration`	2500	25000
Plain DDPG	`sample_batch_size`	20	20
	`train_batch_size`	512	512
	`learning_starts`	5000	5000
	`buffer_size`	100000	100000
	`critics_hidden`	[256, 256]	[256, 256]
TD3	`sample_batch_size`	20	20
	`train_batch_size`	512	512
	`critics_hidden`	[256, 256]	[256, 256]
	`learning_starts`	5000	5000
	`pure_exploration_steps`	5000	5000
	`buffer_size`	100000	100000

Table 6. Variables set to constant values across all RL methods for all environments

Variable	Value set in all RL methods
# worker threads	8
# envs per worker	8
`gamma`	0.99
MLP hidden layers	[400, 300]
